# Clinical and Biological Remission With Tezepelumab: The Real‐World Response in Severe Uncontrolled Asthma

**DOI:** 10.1111/all.16590

**Published:** 2025-05-14

**Authors:** Jessica Gates, Faizan Haris, Francesca Cefaloni, Paniz Khooshemehri, Linda Green, Mariana Fernandes, Louise Thomson, Cris Roxas, Jodie Lam, Grainne d'Ancona, Alexandra M. Nanzer, Jaideep Dhariwal, David J. Jackson

**Affiliations:** ^1^ Guy's Severe Asthma Centre Guy's and St Thomas' NHS Trust London UK; ^2^ School of Immunology and Microbial Sciences King's College London London UK

**Keywords:** asthma, asthma treatment, biologics

## Abstract

**Introduction:**

Tezepelumab is an anti‐TSLP monoclonal antibody approved for the treatment of severe asthma. It has broad downstream anti‐T2 effects, offering the prospect of biological remission. Real‐world data on clinical remission rates with tezepelumab is lacking, and the relationship between clinical and biological remission is unclear. Finally, the effectiveness of tezepelumab in patients who have failed to respond to existing biologic therapies is unknown.

**Methods:**

Clinical and biomarker data from adults with severe asthma treated with tezepelumab in a real‐world setting was analyzed. Clinical outcome measures including clinical remission were recorded along with rates of biological remission (defined as blood eosinophil count < 300 cells/mcL and FeNO < 25 ppb).

**Results:**

One hundred seventy‐five patients were included. 98/175 (56%) had switched from another biologic. Following tezepelumab initiation, the exacerbation rate decreased from 3.1 (2.5) to 0.8 (1.4), with 59% of patients remaining exacerbation‐free at 1 year. 54% achieved an ACQ score < 1.5. Clinical remission at 1 year was observed in 36%, with a rate of 55% in T2‐high patients versus 19% in T2 low patients. The clinical response in biologic‐naïve and biologic switch patients was similar. FeNO declined from 41 ppb (24–76) to 24 ppb (16–38) and BEC fell from 300 cells/μL (60–610) to 180 cells/μL (105–320) (both *p* < 0.001). 38% achieved biological remission. 15% attained both clinical and biological remission.

**Conclusion:**

Tezepelumab led to substantial clinical improvements and clinical remission in up to 55% of T2‐high patients with severe asthma. A disconnect between clinical and biological remission was observed. The long‐term significance of residual T2 inflammation on tezepelumab is unknown.

## Graphical abstract

This study analyzed the clinical and biomarker data from 175 adults with severe asthma treated with the anti‐TSLP monoclonal antibody tezepelumab. Following tezepelumab initiation, the exacerbation rate decreased, with 59% of patients remaining exacerbation‐free at 1 year. Asthma symptom control as measured by ACQ‐6 significantly improved after 4 weeks of tezepelumab, with a further numeric improvement in ACQ‐6 at 6 months of treatment. Rates of clinical remission were significantly greater in patients with an elevated FeNO at baseline and in patients who were composite T2 biomarker high (BEC ≥ 300 cells/μL and FeNO ≥ 50 ppb). *Abbbreviations*: μL, microliter; ACQ‐6, six‐item asthma control questionnaire; BEC, blood eosinophil count; FeNO, fractional exhaled nitric oxide; ppb, parts per billion; TSLP, thymic stromal lymphopoietin.

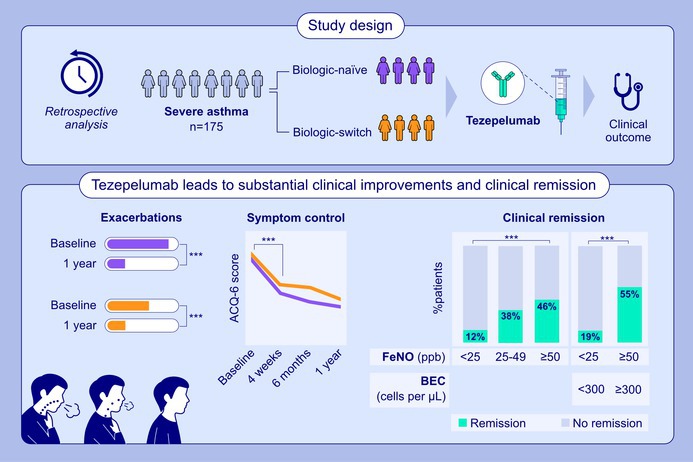

## 1 Introduction

Severe asthma is a heterogenous disease associated with significant morbidity. Randomised controlled trials and real‐world studies have demonstrated that treatment with biologic therapies targeting the type 2 (T2) inflammatory cascade results in significant improvements in exacerbation frequency, symptom scores, and lung function in patients with severe asthma. More recently, a more ambitious response target of clinical remission has become an accepted goal of treatment [1, 2, 3, 4, 5, 6, 7]. Whilst the definition of clinical remission continues to be debated, it is generally agreed that an absence of exacerbations and oral corticosteroid use, along with stability of lung function and good symptom control, are needed.

Tezepelumab is a monoclonal antibody directed against the epithelial‐derived cytokine thymic stromal lymphopoietin (TSLP) preventing binding to its heterodimeric receptor. TSLP acts as an alarmin following perturbation of the airway epithelium and has been identified to play a role in the pathogenesis of asthma through its effects on a wide range of effector cells of the innate and adaptive immune response and activation of multiple downstream inflammatory pathways [8]. In contrast to other asthma biologics, tezepelumab offers the prospect of broad anti‐T2 suppression. In addition, tezepelumab has demonstrated significant clinical improvements in individuals with lower levels of T2 inflammation, becoming the first biologic approved for use in severe asthma without specific T2 biomarker criteria [9].

To date, the main efficacy data for tezepelumab derives from the phase 3 NAVIGATOR study; however, it is recognised that due to strict eligibility criteria and recruitment from non‐specialist asthma centres, the subjects in clinical trials do not accurately reflect the patients with severe asthma prescribed these therapies in the real world. Currently, there is a dearth of data regarding the effectiveness of tezepelumab in a large real‐world cohort of severe asthma patients. Consequently, a clear understanding of how achievable remission is in the real world and which baseline characteristics are associated with an increased or reduced ability to achieve clinical remission with tezepelumab is lacking. Importantly, whether patients who have failed one of the more targeted anti‐IL‐5/5R mAbs improve following a switch to the broader anti‐TSLP approach is unknown. Finally, it remains unknown whether patients who achieve clinical remission also invariably achieve biological remission, or whether there is a disconnect between clinical and biological remission on tezepelumab.

Using a large, well‐characterised cohort of patients with uncontrolled severe asthma treated with tezepelumab at Guy's Severe Asthma Centre, London, UK, we report our findings in relation to these clinically important questions.

## 2 Methods

Sequential adult patients with severe asthma who commenced treatment with tezepelumab between January 2023 and February 2024 under the care of Guy's Severe Asthma Centre, Guy's and St Thomas' NHS Trust, London, UK, were identified for this retrospective analysis. This included patients who were biologic‐naive, as well as patients who had switched from another biologic treatment due to inadequate response. The decision to start tezepelumab was made at the regional severe asthma multidisciplinary meeting which included three specialist asthma physicians. To meet eligibility criteria for tezepelumab according to NICE criteria, patients had to have experienced 3 or more exacerbations in the previous year or be taking maintenance oral corticosteroids [10]. To be included in this analysis, patients must have completed a minimum of 6 months treatment with tezepelumab (210 mg dose every 4 weeks). Before starting tezepelumab, adherence to high dose inhaled and oral corticosteroid therapy was confirmed using ICS/LABA prescription records and paired serum prednisolone and cortisol measurement. Approval to report observational clinical outcome data of this cohort was granted by the Guy's and St Thomas' NHS Ethics Committee (ref 11,999). All the patients in this cohort have additionally previously provided written consent as part of the UK Severe Asthma Registry (Ethics ref. 15/NI/0196).

Clinical outcome measures including forced expiratory volume in litres and as a percentage of predicted (FEV1%), Asthma Control Questionnaire 6 (ACQ‐6) score, asthma exacerbation rate, and maintenance oral corticosteroid (mOCS) dose were retrieved from the electronic patient record at four time points: initiation of tezepelumab, 4 weeks, 6 months and 1 year post‐initiation of tezepelumab therapy. For the patients who were switched from another biologic therapy, relevant clinical characteristics (e.g., blood eosinophil count prior to their previous biologic treatment) were recorded. FeNO and blood eosinophil counts were also recorded.

Clinical remission was defined as an ACQ6 score of ≤ 1.5, elimination of exacerbations, cessation of mOCS for asthma and stable lung function at 1 year [11]. Stable lung function was defined as a change in FEV1 not greater than 5% from the baseline measurement. Biological remission was defined as a FeNO of < 25 ppb and a blood eosinophil count of < 300cells/mcL after 1 year of treatment. Complete remission on treatment was defined as achieving both biological and clinical remission.

Some data is missing in a small number of patients due to either virtual clinical review or missed clinic appointments. Patients with any missing remission criterion at the 1 year timepoint were assumed not to have achieved clinical or biological remission, even if they met all the other remission criteria.

### 2.1 Statistical Analysis

Data were analysed and figures were generated using Graphpad Prism (GraphPad Software, CA, version 9). Results of normally distributed data are reported as mean ± standard deviation (SD) and non‐normally distributed data are reported as median (interquartile range).

Parametric variables were compared using *t*‐tests (paired or independent) or ANOVA (groups); non‐parametric data by Mann–Whitney *U* test (unrelated), Wilcoxon Signed Rank (related) or Kruskal‐Wallis (groups). Categorical variables were analyzed by Chi‐Square test or Fisher's exact test as appropriate. Differences were considered significant at *p* < 0.05.

Predictors of clinical and biological remission were identified from a pre‐determined list of baseline characteristics. Univariate analysis (using methods as above) was used to initially analyze these criteria. Any variables with a *p* ≤ 0.2 were entered into the multivariate logistic regression model. Variables from this model with a *p* value of < 0.05 have been reported with odds ratios (OR) and 95% confidence intervals (CI). The positive predictive value of the model and the goodness of fit using the Hosmer–Lemeshow test are reported.

## 3 Results

One Hundred Seventy‐Five sequential patients (mean age 49.6, 57% female) who had completed a minimum of 6 months treatment with tezepelumab were included in this analysis. 118/175 (67%) of these patients had completed a year of treatment with tezepelumab. 98/175 (56%) had switched from another biologic treatment due to inadequate response, including declining lung function, ongoing exacerbations, and/or requirement for mOCS. Full baseline clinical and phenotypic characteristics are reported in Table 1. Clinical outcome measures for the 118 patients who have completed 1 year of tezepelumab treatment can be found in Table S1.

**TABLE 1:** Baseline patient characteristics at tezepelumab initiation.

	*n* = 175
Female *N* (%)	101 (57%)
Age	49.6 (16.6)
Mean (SD)	
BMI mean (SD)	29.2 (7.6)
Child‐onset asthma *N* (%)	87 (50%)
Atopy *N* (%)	122 (70%)
Nasal Polyposis *N* (%)	68 (39%)
Current smokers *N* (%)	6 (3%)
Ex‐smokers *N* (%)	31 (18%)
Severe exacerbation rate in the previous 1 year Mean (SD)	3.1 (2.5)
ACQ‐6 Mean (SD)	2.55 (1.29)
High dose ICS (2000 mcg BDP or equivalent) *N* (%)	175
Maintenance OCS requirement *N* (%)	43 (24%)
mOCS dose (mg) Mean (SD)	11 (7.8)
Previous biologic *N* (%)	98 (56%)
Switch from Anti‐IL‐5/5R therapy	82 (84%)
Switch from Anti‐IgE therapy	12 (12%)
Switch from Anti‐IL‐4R therapy	4 (4%)
Peak blood eosinophil count in the previous 1 year (×10^9^/L) median (IQR)	0.30 (0.06–0.62)
Peak blood eosinophil count pre first biologic (×10^9^/L)^a^ median (IQR)	0.48 (0.22–0.79)
FENO (ppb) median (IQR)	40 (25–76)
Total IgE (kU/L) median (IQR)	256 (71–878)
FEV1 (L) mean (SD)	2.10 (0.84)
FEV1% predicted mean (SD)	70% (22.3)

*Note:* Atopy was defined as sensitisation to ≥ 1 aeroallergen.

Abbreviations: ACQ‐6, Asthma Control Questionnaire 6; BMI, body mass index; FeNO, fractional exhaled nitric oxide; FEV1, forced expiratory volume in 1 s; ICS, inhaled corticosteroids; IQR, interquartile range; OCS, oral corticosteroid; ppb, parts per billion; SD, standard deviation.

^a^ Peak eosinophil count in the year prior to anti IL‐5/5R or tezepelumab if biologic naïve.

### 3.1 Asthma Exacerbations

**FIGURE 1:**
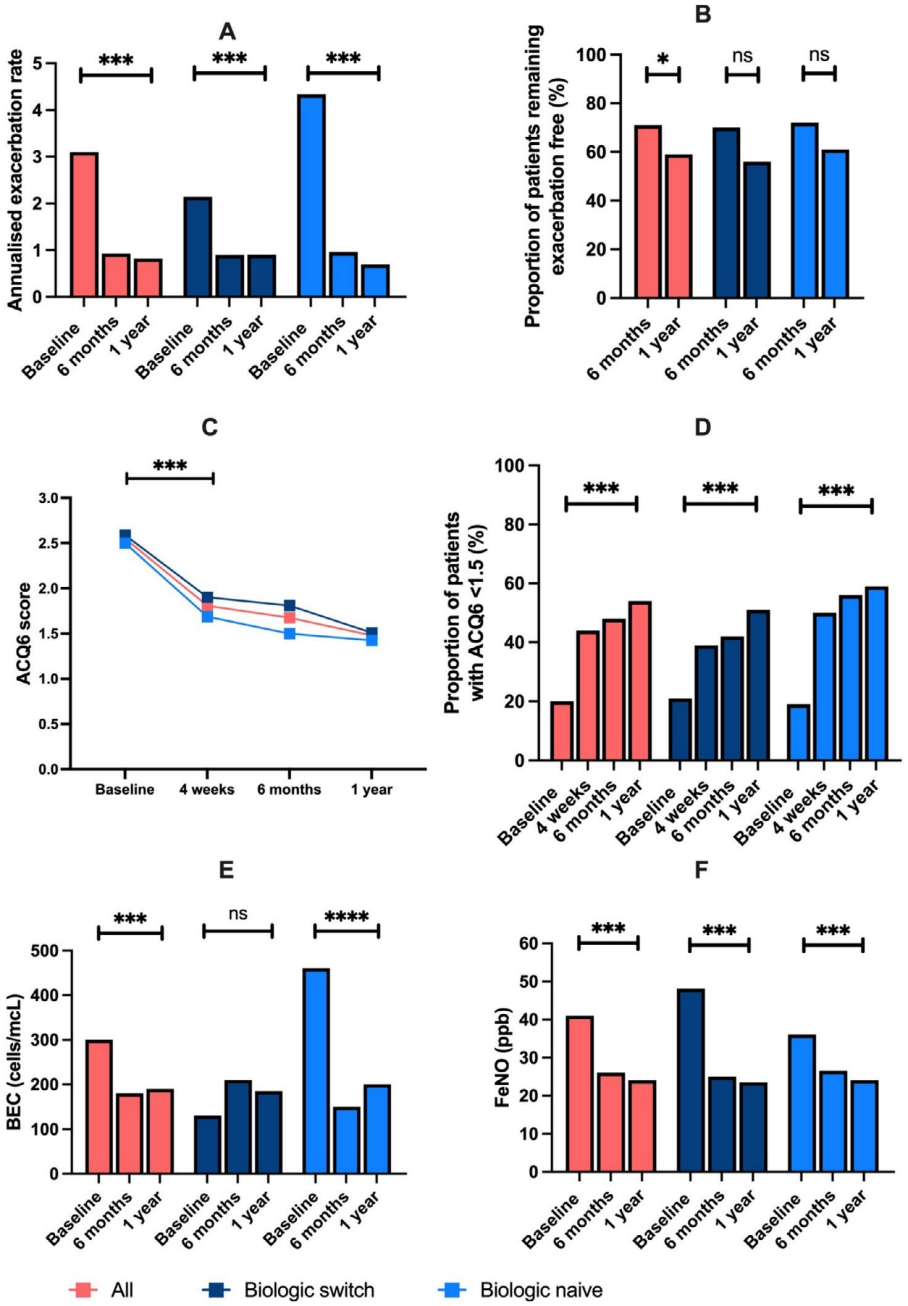
Clinical outcome measures following tezepelumab initiation at 6 months and 1 year. AER (A), proportion of patients remaining exacerbation‐free at 6 months (B), ACQ‐6 score (C), proportion with an ACQ6 score of < 1.5 (D), median BEC (E) and Median FeNO (F) are shown at baseline and after 6 months' treatment with tezepelumab. **p < 0.01, ***p < 0.001, ****p < 0.0001.

The mean annualised exacerbation rate (AER) improved from 3.1 ± 2.5 to 0.9 ± 1.7 at 6 months and to 0.8 ± 1.4 at 1 year (both *p* < 0.001) (Figure 1A). Overall, 59% of patients remained exacerbation‐free at 1 year (Figure 1B). These outcomes were independent of previous biologic use, with a similar exacerbation rate at 1 year and a similar proportion remaining exacerbation‐free irrespective of whether they had failed another biologic prior to tezepelumab initiation.

### 3.2 Asthma Symptom Control

Asthma symptom control as measured by ACQ‐6 significantly improved from 2.55 ± 1.29 to 1.80 ± 1.30 after 4 weeks of tezepelumab (*p* < 0.001), with a further numeric improvement in ACQ‐6 to 1.48 ± 1.20 at 6 months of treatment (6 months vs. baseline, *p* < 0.0001) (Figure 1C). There was no significant difference in ACQ‐6 response between biologic‐naïve and biologic‐switch patients (Figure 1C). Compared to baseline, the proportion of those achieving an ACQ‐6 score of < 1.5 significantly improved after 6 months of tezepelumab (Figure 1D), from 20% to 48% (*p* < 0.001). After 1 year, this increased again to 55% (p < 0.001). There was a trend towards more patients achieving an ACQ‐6 score of < 1.5 at 6 months in the biologic‐naïve group (56%) compared to the biologic‐switch group (42%, *p* = 0.08).

### 3.3 Lung Function

FEV1 (L) significantly improved from 2.11 ± 0.84 L to 2.25 ± 0.8 L at 6 months and 2.37 ± 0.86 L at 1 year (both *p* = 0.0001). The mean (SD) change in FEV1 was +144 (43) mL at 6 months and + 162 (59) mL at 1 year. There were 23 patients who achieved ≥ 500 mL improvement in FEV1 after 6 months.

### 3.4 Requirement for Maintenance OCS

Forty‐three patients required maintenance OCS at the time of starting tezepelumab. Of these, 23 had switched from another biologic. The mean dose of OCS was 11 ± 7.8 mg/day, which significantly improved to 2.9 ± 2.7 mg/day after 6 months of treatment (*p* < 0.0001). By 6 months, only 9/43 patients remained on mOCS due to asthma, with a further 16/43 patients on mOCS for adrenal insufficiency. At 1 year, only 5/32 (16%) of patients remained on mOCS for asthma, with a further 11/32 (34%) of patients on mOCS for adrenal insufficiency.

### 3.5 Change in T2 Biomarkers

Overall, median (IQR) blood eosinophil counts significantly reduced from 300 cells/mcL (60–610) at the start of tezepelumab treatment to 180 cells/mcL (100–300) after 6 months of treatment (*p* < 0.0001), and 190 cells/mcL (100–360) at 1 year (*p* = 0.0007). The baseline BEC in biologic‐naïve patients was higher than the overall cohort at 480 cells/mcL (220–780) due to the residual treatment effect of anti‐IL5/5R therapy and any additional mOCS treatment at baseline in the biologic switch patients. As a consequence of this, the BEC measurements at both 6 and 12 months were numerically (but not statistically) higher than at baseline in this subgroup (Figure 1E).

The level of FeNO significantly fell in all patients in the cohort following initiation of tezepelumab: Median (IQR) FeNO 41 ppb (24–76) at baseline, 26 ppb (17–37) at 6 months, 24 ppb (16–35) at 1 year (*p* < 0.001) (Figure 1F). Overall, the mean (SD) individual fall in FeNO at 6 months was 24[37]ppb, with a significantly higher fall in FeNO observed in the biologic switch vs. biologic‐naïve patients (30[42]ppb vs. 17[30]ppb, *p* = 0.03). At 1 year, the mean individual fall in FeNO from baseline was 34 ppb with no difference between the biologic switch and the biologic‐naïve subgroups.

### 3.6 Clinical Remission Analysis

**FIGURE 2:**
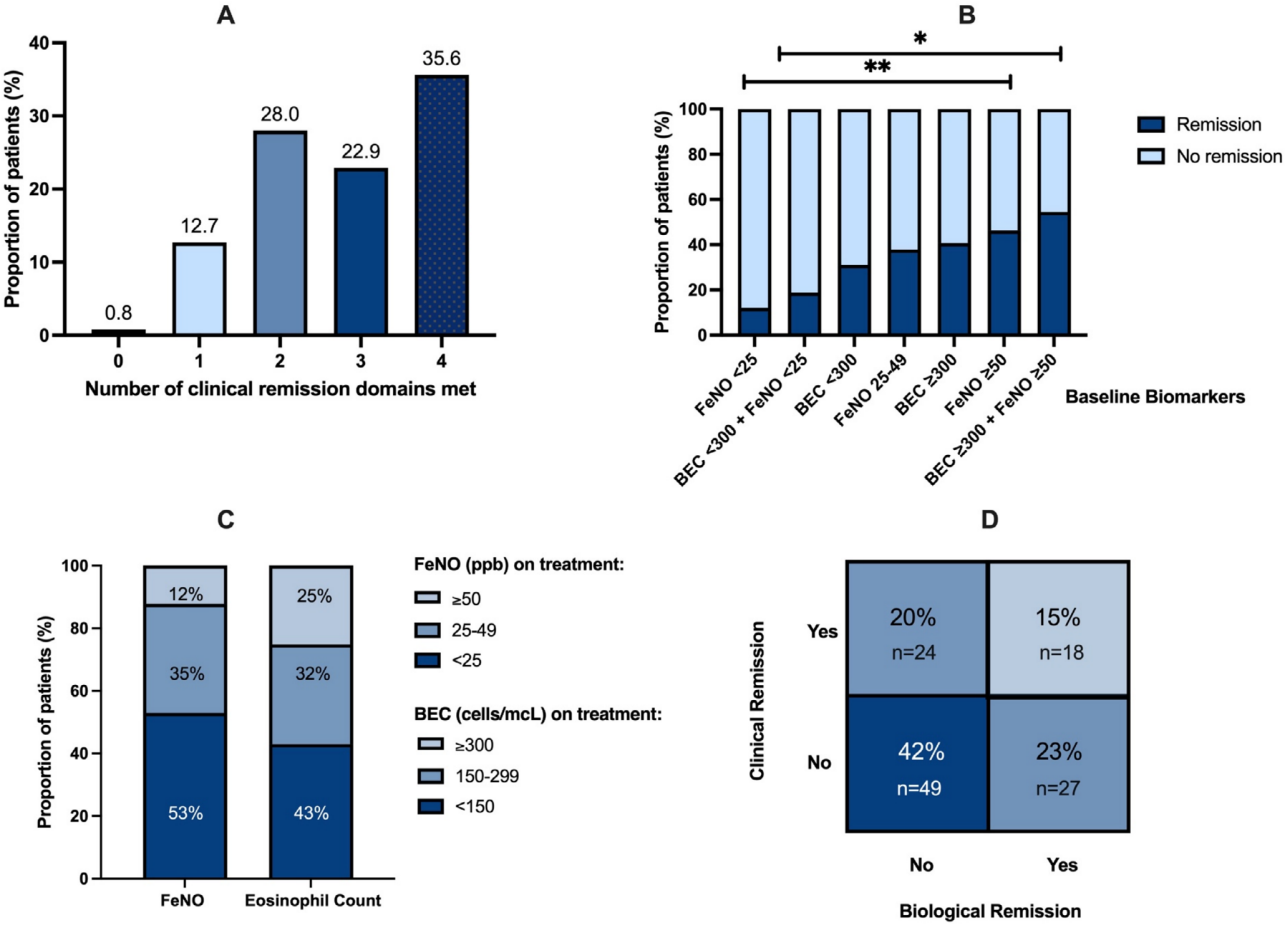
Proportion of patients achieving clinical remission and number of features of remission after 12 months (A); the proportion achieving remission in each baseline T2 biomarker category (*n* = 118) (B); on‐treatment biomarkers after 6 months of tezepelumab (*n* = 175) (C); clinical and biological remission at 12 months with proportions (*n* = 118) (D).

Overall, 42/118 (35.6%) of patients fulfilled the 4‐domain criteria for clinical remission after at least 6 months of tezepelumab. A further 27/118 (22.9%) patients achieved 3 out of 4 domains of remission (Figure 2A). Of the 4 domains at 1 year, 113/118 (96%) were free from maintenance OCS, 85/118 (72%) achieved a stable FEV1, 67/118 (57%) were free from exacerbation, and 65/118 (55%) had an ACQ6 score < 1.5 (Figure S2).

Rates of clinical remission were significantly greater in patients with an elevated FeNO at baseline (46.2% in patients with FeNO ≥ 50 ppb vs. 12% with FeNO < 25 ppb, *p* < 0.01) and in patients who were composite T2 biomarker high (BEC ≥ 300 cells/mcL and FeNO ≥ 50 ppb) vs. composite T2 biomarker low (BEC < 300 cells/mcL and FeNO < 25 ppb) at baseline (54.5% vs. 18.8%, *p* = 0.03) (Figure 2B). Rates of clinical remission in those who were FeNO low (< 25 ppb) and BEC high (≥ 300 cells/mcL) were 0% (0/9) vs. 35% (7/13) in those with high baseline FeNO (≥ 50 ppb) and BEC low (< 300 cells/mcL).

Of the 42 patients who achieved remission at 1 year, 33 (79%) were also in remission at 6 months. Ten patients (23%) who were in remission at 6 months were no longer in remission at 1 year. (Figure S1).

In a univariate analysis, the following baseline clinical characteristics were associated with an increased rate of clinical remission: female gender (*p* = 0.002), lower BMI (*p* = 0.007), adult‐onset asthma (*p* = 0.033), being an ex‐smoker (*p* = 0.004), higher FeNO (*p* = 0.007), and lower ACQ6 (*p* = 0.008) (Table 2). Eleven variables were entered into a multivariate model, and two variables (FeNO and being an ex‐smoker) were retained in the model (Table S2). For each unit increase in baseline FeNO, the patient was 1% more likely to achieve clinical remission. The odds of remission in ex‐smokers was 3.72 times higher.

**TABLE 2:** Phenotypic characteristics of patients achieving clinical remission at 1 year.

	Patients at 1 year (*n* = 118)	Clinical remission (*n* = 42, 36%)	No remission (*n* = 76, 64%)	*p*
Female *N* (%)	68 (58%)	16 (24%)	52 (76%)	0.0014
Age mean (SD)	48.3 (16.5)	51.8 (17.7)	46.5 (15.6)	NS (0.09)
BMI mean (SD)	29.4 (7.7)	26.9 (5.2)	30.8 (8.5)	0.007
Adult‐onset asthma *N* (%)	62 (53%)	28 (45%)	34 (55%)	0.009
Atopy *N* (%)	74 (63%)	25 (34%)	49 (66%)	NS (0.69)
Nasal Polyposis *N* (%)	51 (43%)	22 (43%)	29 (57%)	NS (0.17)
Never smokers *N* (%)	96 (81%)	30 (31%)	66 (69%)	0.049
Ex‐smokers *N* (%)	18 (15%)	12 (67%)	6 (33%)	0.002
Current Smokers *N* (%)	4 (3%)	0 (0%)	4 (100%)	NS (0.29)
Maintenance OCS *N* (%)	32 (27%)	11 (34%)	21 (66%)	NS (0.63)
Biologic switch *N* (%)	73 (62%)	23 (32%)	50 (68%)	NS (0.44)
Peak eosinophil count in the previous 12 months (×10^9^/L) median (IQR)	0.30 (0.04–0.61)	0.39 (0.08–0.77)	0.27 (0.01–0.50)	NS (0.10)
Eosinophil count pre first biologic (×10^9^/L) median (IQR)	0.43 (0.22–0.78)	0.46 (0.12–0.85)	0.43 (0.29–0.71)	NS (0.79)
Baseline FeNO (ppb) median (IQR)	47 (28–84)	70 (36–104)	40 (23–74)	0.007
Total IgE (kU/L) median (IQR)	182 (58–639)	189 (74–1013)	141 (46–491)	NS (0.24)
FEV1 (L) mean (SD)	2.17 (0.83)	2.25 (0.92)	2.12 (0.78)	NS (0.41)
FEV1% Predicted mean (SD)	72% (22)	74% (23.5)	71% (21.2)	NS (0.49)
Exacerbation rate in year prior to Tezepelumab mean (SD)	3.1 (2.5)	2.6 (1.7)	3.4 (2.8)	NS (0.10)
ACQ‐6 mean (SD)	2.58 (1.27)	2.17 (1.12)	2.81 (1.3)	0.008

The multiple logistic regression model demonstrated good overall fit, as indicated by a non‐significant Hosmer–Lemeshow goodness‐of‐fit test (*χ* ^2^ = 10.30, *p* = 0.2447), suggesting that the predicted probabilities were well calibrated to the observed outcomes. The model explained approximately 24.7% of the variance in clinical remission status, as measured by Tjur's *R* ^2^. Discriminative ability was acceptable, with an area under the receiver operating characteristic (ROC) curve (AUC) of 0.7870 (95% CI: 0.7057–0.8683, *p* < 0.0001). Using a classification cutoff of 0.5, the model correctly classified 69.6% of patients, with a sensitivity of 61.3% and specificity of 72.6%.

### 3.7 Biological Remission Analysis

Following initiation of tezepelumab, 53% of patients achieved a FeNO < 25 ppb. Of the remaining 47% who continued to have a FeNO above the normal range, 12% had a FeNO ≥ 50 ppb. Overall, 25% continued to have a BEC ≥ 300 cells/mcL despite at least 6 months of tezepelumab treatment (Figure 2C). At 1 year, 45/118 (38%) achieved biological remission with both a FeNO < 25 ppb and BEC < 300 cells/mcL. Of these, 18/45 also achieved clinical remission, leaving 27/45 (60%) in biological but not clinical remission. Conversely, 18/42 (42.9%) patients in clinical remission also achieved biological remission (Figure 2D).

## 4 Discussion

In this analysis, we set out to report the real‐world clinical response to tezepelumab in a large heterogeneous cohort of adult patients with severe asthma. Firstly, we observed marked clinical responses in all main outcome measures in the majority of patients, irrespective of whether they were previously biologic‐naïve or had switched to tezepelumab from another anti‐T2 biologic due to a suboptimal response; secondly, almost 60% of patients met at least 3 of the 4 clinical remission criteria, with 35.6% achieving all 4 clinical remission domains; thirdly, higher baseline T2 biomarkers increased the likelihood of achieving clinical remission, with rates increasing from less than 20% in composite T2 low patients to 55% in composite T2 biomarker high patients; fourthly that a sizeable minority have ongoing evidence of residual T2 inflammation, including 25% who still have a BEC ≥ 300 cells/mcL, despite treatment with tezepelumab; and finally that the majority of patients who achieve clinical remission do so without having achieved biological remission.

It is interesting to note that despite a more severe asthma cohort than that recruited to the phase 3 NAVIGATOR study (with higher baseline exacerbation rates, higher dependence on maintenance OCS and a large proportion of patients who had failed eosinophil‐directed therapy with an anti‐IL5/5R biologic), the clinical remission rate of 35.6% we observed was higher than the 28.5% reported in the NAVIGATOR subjects [12]. The pooled NAVIGATOR and DESTINATION remission analysis has also noted that those with higher baseline T2 biomarkers are more likely to achieve clinical remission with tezepelumab [12]. It is also of note that these rates are broadly similar to the absolute clinical remission rates reported with other biologics targeting T2 inflammation, including dupilumab (QUEST clinical remission rate of 33.6% in patients on high‐dose ICS at 1 year) [13], and mepolizumab (30% in REDES) [14] Still, based on the baseline exacerbation frequency and mOCS use, our cohort appears more severe. However, caution must be applied to any indirect treatment comparison given key differences in the patient populations recruited to these studies. As can be seen by our own results, overall remission rates with tezepelumab differed dramatically based on the T2 biomarker subgroups. Moreover, definitions of remission vary significantly between trials, with variations of ACQ‐6 cut off (0.75 vs. 1.5) and stability of lung function being the most controversial points. We have chosen an ACQ6 of less than 1.5, as from previous work, it is clear that ACQ6 score is influenced by several extrapulmonary co‐morbidities in severe asthma (e.g., anxiety and obesity) [15].

We have reported an association between being an ex‐smoker and remission with tezepelumab in our cohort. However, caution should be used when assessing this data, as the numbers of ex‐smokers were small, and all except for 1 had either a high FeNO or blood eosinophil count, which could be contributing to their apparent high remission rates. Previous work has shown that those with a smoking history appear to have similar outcomes with tezepelumab to those who have never smoked; however, this finding cannot easily be explained mechanistically, and larger prospective studies are needed to confirm this finding.

Much attention has been given to the clinical trial data suggesting tezepelumab efficacy across the T2/eosinophilic spectrum including in ‘T2 low’ patients. However, it should be noted that in a pooled analysis of the phase 2b PATHWAY and phase 3 NAVIGATOR trials, there was not in fact a statistically significant reduction in exacerbations in the subgroup of patients genuinely lacking evidence of T2 inflammation (i.e., both FeNO < 25 ppb and blood eosinophil count < 150 cells/μL, excluding subjects with suppressed T2 inflammation simply due to maintenance oral corticosteroid therapy) [16]. Our own results confirm that the best clinical response to tezepelumab is seen in patients with elevated levels of T2 inflammation. Overall we observed a remission rate of 55% in composite T2 biomarker high patients compared to a rate of only 18% in T2 composite low individuals, raising further doubt about the efficacy in this group of patients. Definitions of biological remission vary, particularly with regard to using a FeNO of 20 ppb versus 25 ppb. We have chosen to use a cut‐off of 25 ppb, as this is what is used in the NAVIGATOR trial, in order to provide a direct comparison. Although some studies use a blood eosinophil count of < 150 cells/μL, it has previously been shown that the 5%–95% range of eosinophil counts in the healthy adult population is 30–330 cells/μL, hence we have opted to use limit of 300 cells/μL for the purpose of this analysis [17].

In recent years, there has been a renewed focus on clinical remission, however, the significance of biological remission has been far less studied. Key outstanding questions have included whether biological remission is necessary for attainment of clinical remission; and whether there are potentially longer‐term clinical implications of not achieving biological remission? Our results suggest that there appears to be a disconnect between clinical and biological remission in patients treated with tezepelumab. On the one hand, the majority of patients achieving clinical remission continued to have evidence of T2 inflammation (BEC ≥ 300 cells/mcL and/or FeNO > 25 ppb), whilst on the other hand, the majority of patients who fulfilled the biological remission criteria did not achieve clinical remission. In the former group there is a potential concern that subclinical T2 inflammation may permit accelerated lung function decline due to IL‐13 related airways remodelling over a longer period of time. In the latter group there is the possibility that symptoms and exacerbation events are unlikely to respond to any biologic therapy given the absence of T2 inflammation and may reflect chronic airway infection and/or other extrapulmonary causes of ‘asthma’ symptoms.

Our results also reveal the extent of residual T2 inflammation with tezepelumab. Specifically, we observed that 25% of patients still had a BEC ≥ 300 cells/mcL whilst 47% still had a FeNO ≥ 25 ppb (with 12% exceeding a FeNO 50 ppb on tezepelumab). It remains unclear if higher doses of tezepelumab would more completely suppress this T2 inflammation, or whether it primarily reflects the importance of epithelial‐derived cytokines other than TSLP, including IL‐33 and/or IL‐25 [18, 19, 20].

Our analysis represents the first report of tezepelumab effectiveness in patients with severe asthma who have failed to adequately respond to other biologic therapies, in particular those targeting the IL5‐eosinophil pathway. The observation that these individuals had marked improvements in clinical responses following initiation of tezepelumab is mechanistically interesting and is consistent with our previous report of similarly impressive responses to the anti‐IL4R mAb dupilumab [5]. In both groups, the patients appeared to have a T2, but non‐eosinophil dominant, inflammatory phenotype with particularly high baseline FeNO levels. The poor response to the anti‐IL5/5R mAbs in these cohorts was despite BEC counts > 300cells/mcL prior to treatment. This delineation of eosinophil and non‐eosinophil (presumed IL‐13 driven) components of T2 inflammation remains poorly understood but was further revealed in the SHAMAL study in which some eosinophil‐deplete patients treated with benralizumab had evidence of accelerated lung function decline following cessation of maintenance ICS therapy in the context of a rising FeNO [21]. Whether tezepelumab might be more permissive of complete ICS cessation than benralizumab due to its broader anti‐T2 effects is the subject of an ongoing clinical trial (NCT06473779).

Advantages of this analysis are the large, well‐characterised cohort size, the real‐world design allowing inclusion of a heterogeneous group of severe asthma patients (many of whom would not have been eligible for the phase 3 NAVIGATOR study), and the in‐depth analysis of both clinical and biological remission. However, we acknowledge the limitations inherent in any real‐world study lacking a control arm, as well as the retrospective nature of this analysis.

In summary, we report the first large real‐world analysis of tezepelumab effectiveness and clinical remission in severe asthma and in particular in patients who have failed to respond adequately to anti‐IL5/5R therapy. We demonstrate considerable clinical benefits in the majority of biologic‐naïve and biologic‐experienced patients treated with tezepelumab; we reveal significant residual T2 inflammation despite TSLP blockade and in so doing highlight a disconnect between clinical and biological remission on tezepelumab. Finally, it is important to appreciate that despite early enthusiasm about the efficacy of tezepelumab in ‘T2‐low’ patients, less than 20% of this subgroup appear to achieve clinical remission, with the greatest clinical responses in those with the highest baseline levels of T2 inflammation. A substantially effective treatment option for this poorly understood ‘T2‐low’ subgroup remains elusive.

## Conflicts of Interest

J.G. reports payment or honoraria for lectures and educational events from Astra Zeneca and Sanofi. A.M.N. reports payment or honoraria for lectures and educational events from Astra Zeneca, Glaxo Smith Kline, and Sanofi and support for attending conferences from Astra Zeneca, Chiesi, and Napp. L.T. reports payment or honoraria for lectures and educational events and travel support from Astra Zeneca and Teva. GdA reports advisory board work for, been engaged to provide training to HCPs on behalf of, received research grants from, and/or been sponsored to attend an educational meeting by: AstraZeneca, Boehringer‐Ingleheim, Chiesi, GlaxoSmithKline, NAPP, Novartis, Pfizer, Sanofi, Teva. J.D. reports support to attend conferences from Sanofi. C.R. reports honoraria for lectures and conference support from Astra Zeneca. D.J.J. reports research grants to the department from Astra Zeneca, consulting fees from Astra Zeneca, GSK, and Sanofi Regeneron, payments or honoraria for lectures and educational events from Astra Zeneca, GSK, and Sanofi.FC is a recipient of the European Academy of Allergy and Clinical Immunology (EAACI) Research Fellowship 2024 and has received honoraria from Astra Zeneca, GSK, Sanofi, Chiesi, and Doxa Pharma. L.G., M.F., J.L.L., T.M., F.H., and P.K. report no COI.

## Supporting information


Figure S1.

Figure S2.

Table S1.

Table S2.

## Data Availability

The data that support the findings of this study are available on request from the corresponding author. The data are not publicly available due to privacy or ethical restrictions.
