# Supplementary material for: Clinical and Biological Remission With Tezepelumab: The Real‐World Response in Severe Uncontrolled Asthma
Source: Allergy. 2025 May 14;80(6):1669–76. doi: 10.1111/all.16590 (PMC12186586; doi:10.1111/all.16590)
Supplement: Supplementary file 1 — Figure S1. [file ALL-80-1669-s002.docx]

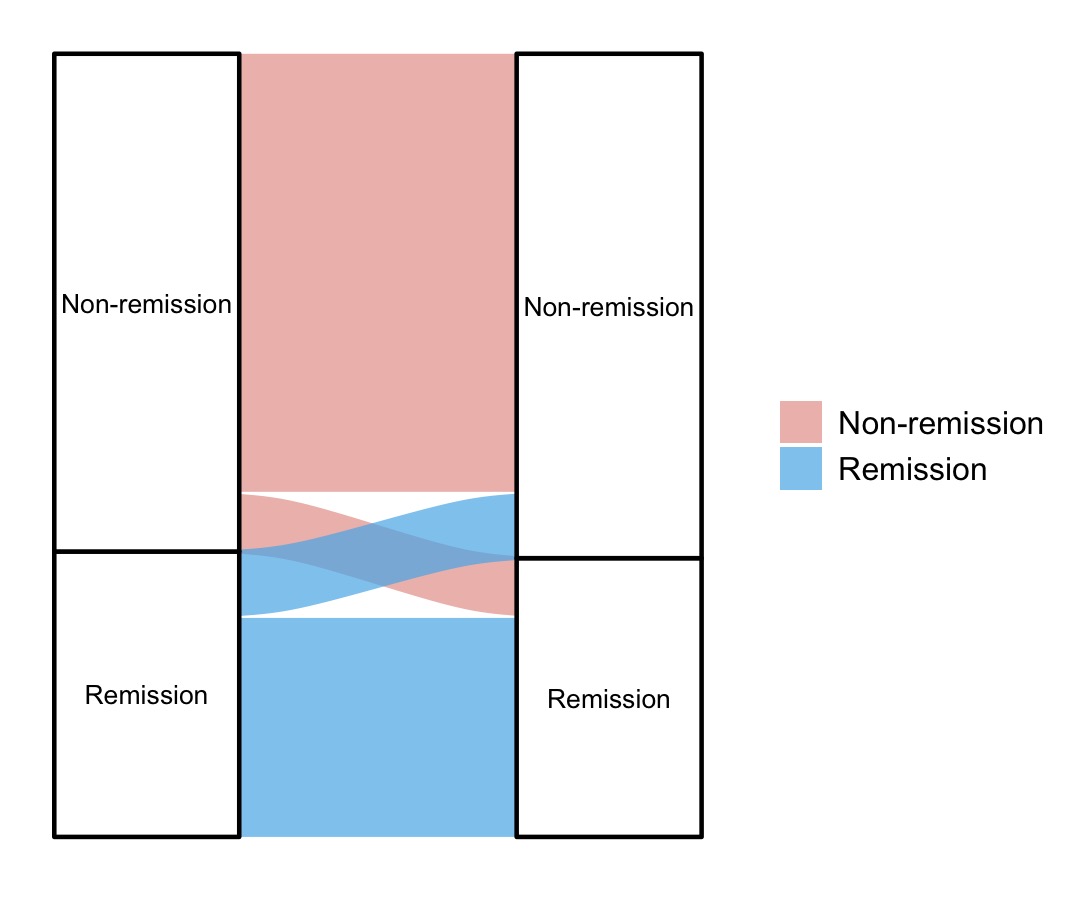


Supplementary Figure 1: Sankey plot demonstrating the movement between the Non-remission or Remission groups at 6 months to 1 year.
