# Supplementary material for: Clinical and Biological Remission With Tezepelumab: The Real‐World Response in Severe Uncontrolled Asthma
Source: Allergy. 2025 May 14;80(6):1669–76. doi: 10.1111/all.16590 (PMC12186586; doi:10.1111/all.16590)
Supplement: Supplementary file 2 — Figure S2. [file ALL-80-1669-s004.docx]

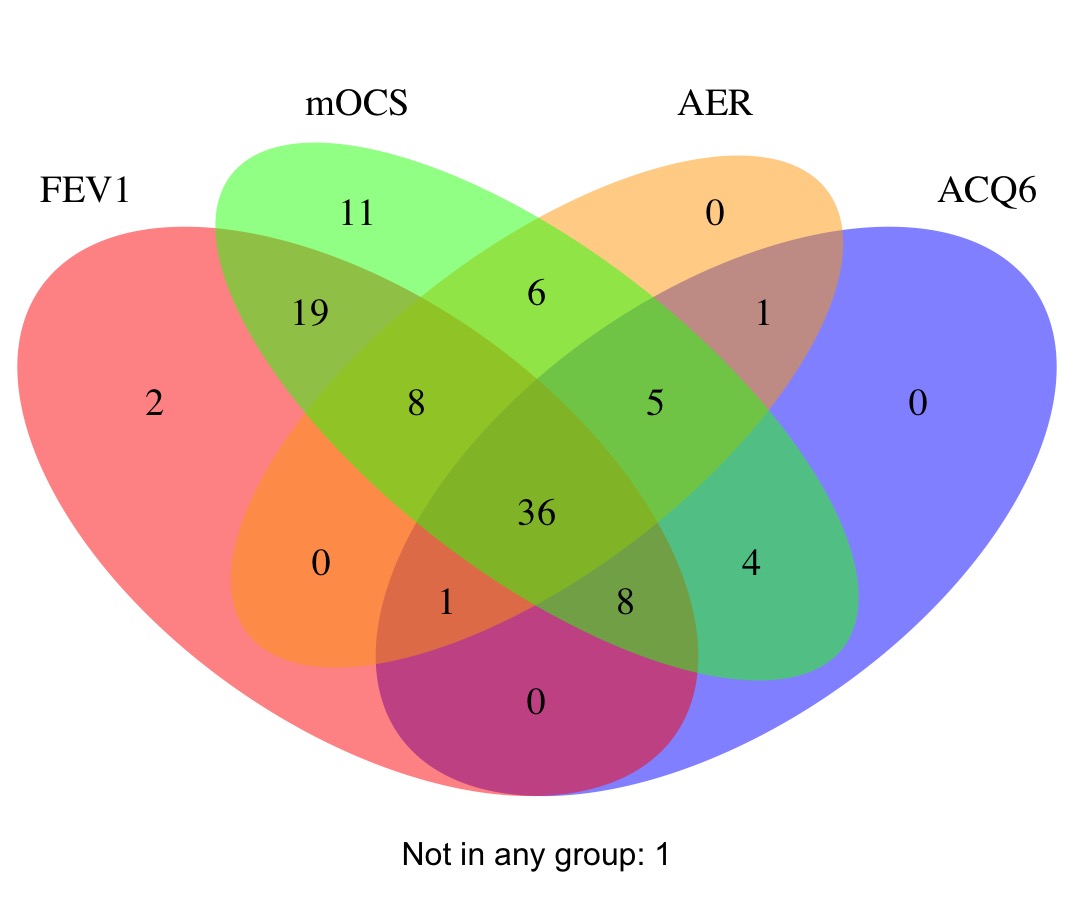


Supplementary Figure 2: Proportion of patients (%) achieving each of the 4 domains of clinical remission at 1 year (n=118)
