# Supplementary material for: Clinical and Biological Remission With Tezepelumab: The Real‐World Response in Severe Uncontrolled Asthma
Source: Allergy. 2025 May 14;80(6):1669–76. doi: 10.1111/all.16590 (PMC12186586; doi:10.1111/all.16590)
Supplement: Supplementary file 3 — Table S1. [file ALL-80-1669-s001.docx]

| N=118 | Baseline | 6 months | 1 year | p value |
| --- | --- | --- | --- | --- |
| AER  Mean (SD) | 3.1 (2.5) | 0.9 (1.5) | 0.8 (1.4) | <0.001 |
| Patients remaining exacerbation free  N (%) | 15 (13%) | 79 (67%) | 67 (57%) | <0.001 |
| ACQ6 score  Mean (SD) | 2.58 (1.27) | 1.62 (0.5-2.5) | 1.48 (1.20) | <0.001 |
| Patients with an ACQ6 score <1.5  N (%) | 23 (19%) | 59 (50%) | 65 (55%) | <0.001 |
| Blood eosinophil count  Median (IQR) | 0.3 (0.04-0.61) | 0.2 (0.11-0.35) | 0.19 (0.10-0.36) | <0.001 |
| FeNO  Median (IQR) | 46.5 (28-83.5) | 25.5 (18-37) | 24 (16-35) | <0.001 |

Supplementary table 1: Clinical outcome measures following tezepelumab initiation at 6 months and 1 year, only in those patients who have completed 1 year of treatment (n=118).
