# Supplementary material for: Clinical and Biological Remission With Tezepelumab: The Real‐World Response in Severe Uncontrolled Asthma
Source: Allergy. 2025 May 14;80(6):1669–76. doi: 10.1111/all.16590 (PMC12186586; doi:10.1111/all.16590)
Supplement: Supplementary file 4 — Table S2. [file ALL-80-1669-s003.docx]

| Variables | OR | p-value | 95% CI |
| --- | --- | --- | --- |
| Baseline FeNO (ppb) | 1.01 | 0.035 | 1.002-1.022 |
| Ex-smoker | 3.72 | 0.039 | 1.106-13.77 |

Supplementary table 2 – Independent predictors of clinical remission.
